# Christianus Fridericus Reufs de virtutibus Caricis arenariæ radicis Sarsaparillæ vires fere superantibus

**Published:** 1785

**Authors:** 


II. Chriftianus Fridericus Reufs *de virtutibus Caricis arenariæ radicis Sarfaparillæ vires fere fuperantibus.* Vide *Novorum actorum phyfico-medicorum Academiæ Cæfareæ Leopoldino-Carolinæ Naturæ Curioforum Tomum feptimum.* 4to. Norimbergæ, 1783.

THE defign of this paper is to recommend the root of the *Carex arenaria* as a fubftitute

ſtitute for that of ſarſaparilla. A decoction of it, the author obſerves, has long been ſucceſsfully employed for this purpoſe in the Pruſſian army; but he has omitted to give us any experiments that might tend to aſcertain the comparative efficacy of the two remedies. His inquiry is confined chiefly to their chemical analyſis; and, as the proportion of the ſpirituous to the watery extract yielded by the Carex arenaria is greater than that afforded by the ſarſaparilla, he is diſpoſed to give the preference to the former for medical purpoſes.

What he ſays of its uſe in Pruſſia is on the authority of Profeſſor Gleditch, whom we find expreſſing himſelf in the following manner on this ſubject, in the Memoirs of the Academy of Sciences at Berlin, for the year 1768:

" There is one ſpecies of *Carex*, commonly " called (by the Germans) *Flugſand*, (*Carex arenaria*, LINN.) which, from the moſt accurate " experiments, appears to be of great uſe in " phyſic. Its freſh root, of which, in ſome " parts of the Marquiſate (of Brandenburgh,) " great quantities may eaſily be collected, has, " when cleaned, ſome reſemblance to that of " ſarſaparilla. . . . . . It has a ſweetiſh, balſamic " taſte; the tinctures, extracts, and other pre-

" parations of it with water, wine, or rectified " spirit, resemble similar preparations of guai- " acum wood: and, in a word, its smell, " taste, effect, and manner of operating, prove " that it possesses virtues infinitely superior to " those of sarsaparilla. For some years past " it has been employed at Berlin, and other " places, in His Majesty's army, with great " success, in lieu of an exotic and expensive " remedy."

Since the publication of Professor Gleditch's observations, this new substitute for sarsaparilla has been the subject of two Dissertations. One of these by Dr. C. G. Meier, *de Carice Arenaria sive Sassaparilla Germanica*, is accompanied with an engraving of the plant, and was printed at Frankfort on the Oder in 1772; the other by Dr. C. F. Merz, *de Caricibus quibusdam medicinalibus Sarsaparillæ succedaneis*, at Erlangen in 1784. In both of these works we meet with an accurate botanic and chemical history of the plant in question; but neither of them seems to relate any thing of importance, from the experience of the authors, concerning its effects in diseases. Dr. Merz supposes two other species of Carex, viz. the *C. disticha*, and *C. hirta*, to be of equal efficacy with the *C. arenaria*.

*arenaria.* — As each of thefe fpecies is common in Great Britain, we fhall hope foon to fee their medicinal properties more fatisfactorily afcertained.

